# Editorial: Malaria Targeting Toolkit: Host-Parasite Interactions

**DOI:** 10.3389/fcimb.2021.795494

**Published:** 2021-11-17

**Authors:** Tamasa Araki, Jing-wen Lin, Miguel Prudêncio, Deirdre A. Cunningham, Takeshi Annoura

**Affiliations:** ^1^ Department of Parasitology, National Institute of Infectious Diseases (NIID), Tokyo, Japan; ^2^ State Key Laboratory of Biotherapy, West China Hospital, Sichuan University and Collaborative Innovation Center for Biotherapy, Chengdu, China; ^3^ Instituto de Medicina Molecular, Faculdade de Medicina, Universidade de Lisboa, Lisboa, Portugal; ^4^ The Francis Crick Institute, London, United Kingdom

**Keywords:** *Plasmodium*, malaria, drug development, immune response, parasite virulence, host-parasite interactions

Malaria remains a serious threat to public health worldwide. Protozoan *Plasmodium* parasites, the etiological agents of malaria, are transmitted to their mammalian hosts by *Anopheles* mosquitoes. Despite decades of collective efforts, malaria remains one of the most devastating infectious diseases in the globe, with more than half of the world’s population at risk of infection and more than 400 thousand annual deaths. Malaria is a complex disease with a broad spectrum of symptoms, ranging from mild forms to severe pathology, the latter occurring in patients with life-threatening anemia, cerebral malaria, metabolic acidosis, and multiorgan system failure. In order to provide new insights into the complex interplay between the parasite and its mammalian host during the liver or blood stages of *Plasmodium* infection, the identification of key parasite or host molecules that interact with each other or that critically influence the outcome of infection, and the understanding of immune responses to infection, remain a priority (**Figure 1**). The present Research Topic consists of 11 papers, including 3 reviews, 1 perspective, 1 brief research report, and 6 original research articles.

**Figure 1:**
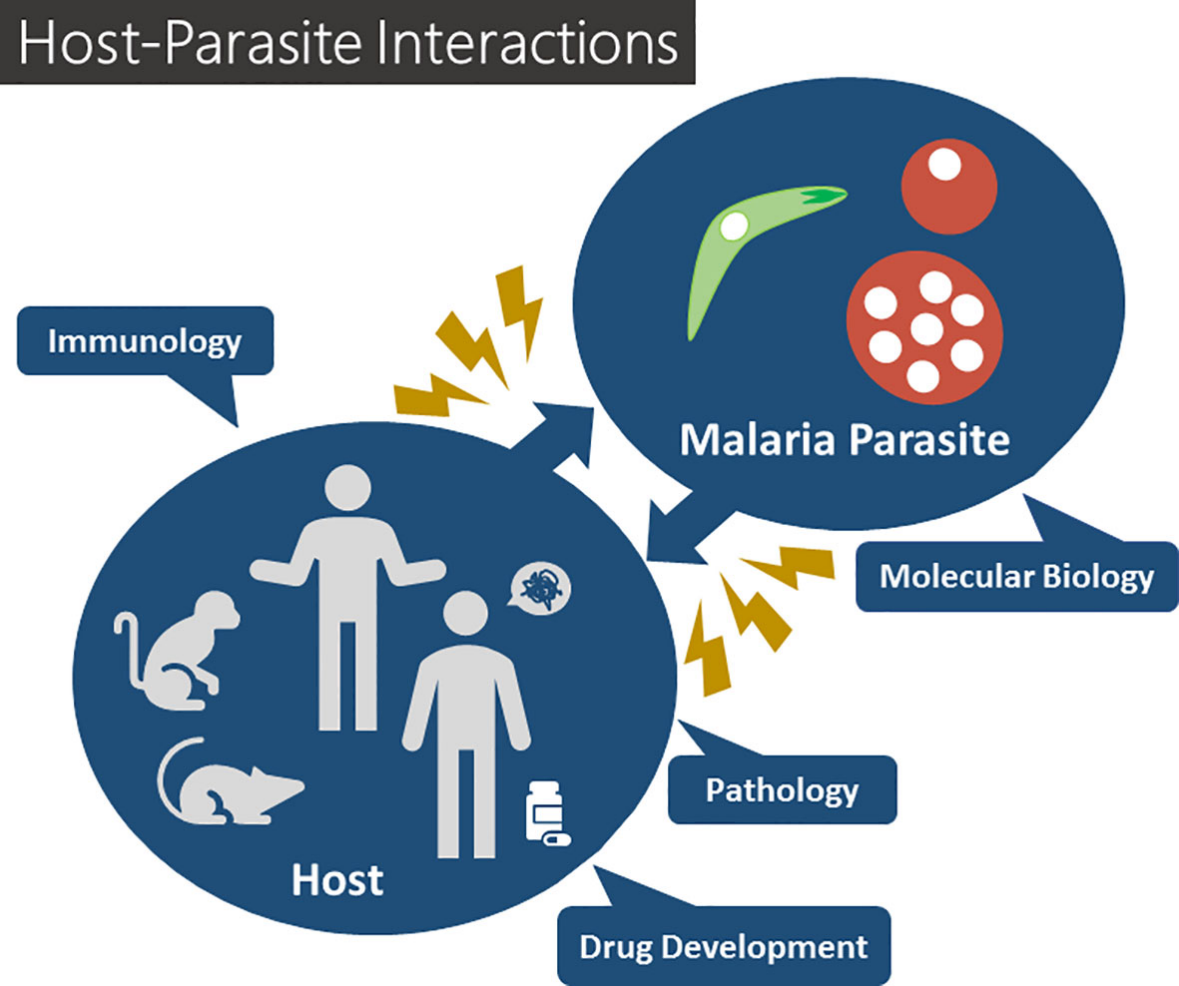
Host-parasite interactions in malaria.

Dhangadamajhi and Singh described the role of plasma sphingosine 1-phosphate (S1P), recently shown to play an important role in the regulation of various physiological processes related to malaria pathogenesis. S1P is a bioactive lipid intermediate of sphingolipid metabolism that exists in intracellular (such as red blood cells and platelets) and circulating extracellular pools, with each pool having a different function. In this review, the authors summarised the significance of each of these pools in the context of malaria. They also discussed how the S1P content in the erythrocytes could be channelled towards future therapeutic opportunities for malaria.

An original research article revealed the role of the *Plasmodium berghei* quiescin sulfhydryl oxidase (PbQSOX), which is present in various eukaryotic species and catalyses the insertion of disulphide bonds into unfolded and reduced proteins. Zheng et al. show that PbQSOX is most abundantly expressed in ookinetes, and localizes in the cytoplasm and surface of ookinetes. Further, the authors showed that mosquitoes fed on Δpbqsox parasite-infected mice displayed a significant reduction in ookinete and oocyst numbers compared to those fed on wild-type parasite-infected mice. Furthermore, both polyclonal antisera and a monoclonal antibody against recombinant PbQSOX exhibited substantial transmission-blocking activities *in vitro* and in the mosquito feeding assays. These findings identified QSOX as a potential target for blocking parasite transmission.

Another article discussed the potential of chimeric *Plasmodium falciparum* (Pf) parasites expressing the *P. vivax* (Pv) circumsporozoite protein (CSP) as a tool for the investigation of vaccines against Pv. Chimeric rodent malaria parasites expressing Pf and Pv CSP have previously been used in the preclinical evaluation of CSP-based pre-erythrocytic vaccines. Miyazaki et al. have now generated chimeric *P. falciparum* parasites expressing both the endogenous PfCSP and the heterologous PvCSP. The authors showed that immunization of mice with these Pf-PvCSP sporozoites elicited the production of antibodies against the repeat regions of both PfCSP and PvCSP, supporting the use of the newly generated parasite line in the optimization of vaccines targeting PvCSP.

A review by He et al. discussed the ‘conflicting’ roles exhibited by type I interferons (IFN-ls) during malarial infections. IFN-Is are important cytokines that play a role in various infections, autoimmune diseases, and cancer. Both the liver and the blood stages of malaria parasites can stimulate IFN-I responses in their vertebrate host. The host’s genetic background greatly influences IFN-I production during *Plasmodium* infections as well. Consequently, the effect of IFN-Is on parasitemia and disease symptoms are highly variable, depending on the combination of the parasite species/strains and the host. This review also summarized the major findings and progress in ligand recognition, signalling pathways, functions, and regulation of IFN-I responses during mammalian infections by *Plasmodium* parasites.

An article by Ito et al. showed that novel proteins of apical organelles in merozoites are involved in erythrocyte invasion by *Plasmodium falciparum*. The authors generated a panel of monoclonal antibodies against *P. falciparum* schizont-rich parasites and screened the antibodies using immunofluorescence assays. Five of the monoclonal antibodies were then used in immuno-affinity experiments to purify their target antigens. Afterwards, liquid chromatography-tandem mass spectrometry (LC-MS/MS) was used to identify these antigens. The authors further expand on a novel complex that was identified by immunoprecipitation, composed of *P. falciparum* rhoptry-associated membrane antigen (PfRAMA) and *P. falciparum* rhoptry neck protein 3 (PfRON3) and identified a region spanning amino acids Q221-E481 of PfRAMA that can associate with PfRON3 in the immature schizonts.

A mini-review provided exciting new insights on the cross-talk between the *Plasmodium* parasite and its mammalian host. Voorberg-van der Wel et al. zoomed in on new technologies using monkey malaria and other malaria parasite model systems that can provide the tools necessary to investigate host-parasite interactions of relapsing parasites. The authors proposed that learning more about hypnozoite and host cross-talks should help identify factors that promote the activation of these dormant parasite reservoirs, leading to disease relapses, and may contribute to the identification of new tools aimed at eventually achieving malaria eradication.

A perspective topic authored by Oresegun et al. discussed underlying pathophysiological processes leading to a serious malaria infection. The authors described zoonotic malaria caused by *P. knowlesi* and proposed the use of this parasite as a model system specifically for severe malaria. Subsequently, a method to generate long-read third-generation *Plasmodium* genome sequence data from archived clinical samples was developed using the MinION platform. This research then developed a representative translational model for severe malaria that was informed by clinically relevant parasite diversity due to its ability to exploit the duality of *P. knowlesi* as a laboratory model and human pathogen.

Another article by Taku et al. discussed how the transport protein N-myristoylated adenylate kinase 2 (PfAK2) is transported and regulated by Rab GTPases from the endoplasmic reticulum (ER) to the cell’s surface. Previously, the same group reported that the export of PfAK2, which lacked the PEXEL (*Plasmodium* export element) motif, was regulated by PfRab5b GTPase (Ebine et al., 2016). These authors now expand their findings and demonstrate that PfRab5b and the associated GTPases are involved in trafficking PfAK2 and PEXEL-positive Rifin from the ER. Two membrane trafficking GTPases, PfArf1 and PfRab1b, were also coimmunoprecipitated with PfRab5b and subjected to mass spec analysis. Their results showed that PfArf1 and PfRab1b colocalised with PfRab5b adjacent to the ER marker, PfBiP. Furthermore, results from the mutant analysis of PfArf1 and Rab1b suggest that PfArf1 was extensively involved in the transportation of PfAK2. The data showed that PfArf1 and PfRab1b are also involved in RIFIN transport, thereby indicating the sequential roles of PfArf1 and PfRab1b in cargo selection. The research is the first report on cargo selection of PfAK2 on the ER’s subdomain by PfArf1.

The molecular mechanisms underlying the protection against malaria offered by the heterozygous carriage of the haemoglobin S mutation (HbAS), or sickle cell trait, are still poorly understood. The original article by Chauvet et al. investigated the proteome and phosphoproteome of red cell membrane extract from *P. falciparum*-infected and uninfected red blood cells (RBCs) from heterozygous S carriers (HbAS) and homozygous non-mutant (HbAA) donors. The authors identified proteins whose quantity or phosphorylation state varied according to infection status and/or host haemoglobin genotype. Changes due to the S trait, to *P. falciparum* infection, or both were highlighted. Proteins identified included erythrocyte membrane transporters, skeletal proteins, and parasite proteins such as RESA or MESA. Phosporylation state may influence multiple functions such as membrane interactions, nutrient transport, cytoadherence. This is the first report of phosphorylation patterns in HbAS RBCs, infected or uninfected, and thus provides clues to understanding the molecular mechanisms of malaria resistance and importantly also identified alterations in phosphorylation state solely due to the carrier status.

The original research article by Ikeda et al. employed a novel genetic tool, *P. berghei* mutator (PbMut), whose base substitution rate is 36.5 times higher than that of wild-type parasites, to analyse a mutant parasite line (PbMut-PPQ-R-P9) with reduced susceptibility to piperaquine (PPQ), unveiling a new mechanism of drug resistance in malaria parasites. Whole-genome sequence analysis of PbMut-PPQ-R-P9 clones revealed that eight nonsynonymous mutations were conserved in all clones, including N331I in PbCRT, which encodes the chloroquine resistance transporter (CRT). Furthermore, the PPQ susceptibility and growth rates observed in genome-edited parasites (PbCRT-N331I) were significantly lower than those of PbMut-PPQ-R-P9, implying that additional mutations in PbMut-PPQ-R9 parasites compensate for the fitness cost of the PbCRT (N331I) mutation and contribute to reduced PPQ susceptibility. In summary, PbMut served as a novel genetic tool for predicting gene mutations responsible for drug resistance. Nevertheless, further studies on PbMut-PPQ-R-P9, which are likely to reveal genetic changes that compensate for fitness costs owing to drug resistance acquisition, should be conducted.

This original research article clarifies *Plasmodium*’s mechanism of mRNA quality control and export using RNA-binding proteins of malaria parasites. Previous research had shown that nuclear poly(A) binding protein 2 (NAB2), THO complex subunit 4 (THO4), nucleolar protein 3 (NPL3), G-strand binding protein 2 (GBP2), and serine/arginine-rich splicing factor 1 (SR1) are involved in nuclear mRNA export (Eshar et al., 2015). Niikura et al. now found that NAB2 and SR1, but not THO4, NPL3, or GBP2, influence the asexual development of malaria parasites. In contrast, GBP2, but not NPL3, is involved in male and female gametocyte production; therefore, the authors focus on the contribution of GBP2 and NAB2 to the sexual and asexual development of malaria parasites. They found out that GBP2 interacts with ALBA4, DOZI, CITH, and with the phosphorylated adapter RNA export (PHAX) domain-containing protein, which plays a role in translational repression. Alternatively, NAB2 interacts with transportin and binds directly to 143 mRNAs, including those encoding 40S and 60S ribosomal proteins.

In summary, the articles in this Frontier’s Research Topic “Malaria Targeting Toolkit: Host-Parasite Interactions” bring important new data as well as thoughtful insights about host-parasite interactions, parasite virulence, immune responses and molecular mechanisms of host resistance to parasites, and parasite resistance to drugs. Novel tools and methodologies are being developed, state-of-the-art techniques expanded, and many new insights gained, which will inform future malaria drug and vaccine development efforts.

## Author Contributions

TAr and TAn prepared the draft. TAr, JL, MP, DC, and TAn revised the draft and approved the final version of the draft. All authors contributed to the article and approved the submitted version.

## Funding

This work was supported in part by a grant for Research on Emerging and Re-emerging Infectious Diseases from the Japan Agency for Medical Research and Development (AMED) (JP21fk0108096j0303, JP21fk0108138j0602, and JP21fk0108139j3002 to TAn), a grant from the Japan Society for the Promotion of Science (JSPS) (KAKENHI JP21K06999 to TAn) and a grant from The Chemo-Sero-Therapeutic Research Institute (90011100) to TAn. This work was supported in part by the National Natural Science Foundationof China (No. 31800772) to J-wL.

## Conflict of Interest

The authors declare that the research was conducted in the absence of any commercial or financial relationships that could be construed as a potential conflict of interest.

## Publisher’s Note

All claims expressed in this article are solely those of the authors and do not necessarily represent those of their affiliated organizations, or those of the publisher, the editors and the reviewers. Any product that may be evaluated in this article, or claim that may be made by its manufacturer, is not guaranteed or endorsed by the publisher.
